# Incidence of Platelet Dysfunction by Thromboelastography–Platelet Mapping in Children Supported with ECMO: A Pilot Retrospective Study

**DOI:** 10.3389/fped.2015.00116

**Published:** 2016-01-06

**Authors:** Arun Saini, Mary E. Hartman, Brian F. Gage, Ahmed Said, Avihu Z. Gazit, Pirooz Eghtesady, Umar S. Boston, Philip C. Spinella

**Affiliations:** ^1^Department of Pediatrics, The University of Tennessee Health Science Center, Memphis, TN, USA; ^2^Department of Pediatrics, Washington University in St. Louis, St. Louis, MO, USA; ^3^Department of Medicine, Washington University in St. Louis, St. Louis, MO, USA; ^4^Department of Cardiothoracic Surgery, Washington University in St. Louis, St. Louis, MO, USA

**Keywords:** platelet mapping, thromboelastography, ECMO, anticoagulation, platelet dysfunction, heparin, ECMO-induced coagulopathy

## Abstract

**Background:**

Bleeding complications are common and decrease the odds of survival in children supported with extracorporeal membrane oxygenation (ECMO). The role of platelet dysfunction on ECMO-induced coagulopathy and resultant bleeding complications is not well understood. The primary objective of this pilot study was to determine the incidence and magnitude of platelet dysfunction according to thromboelastography (TEG^®^)–platelet mapping (PM) testing.

**Methods:**

Retrospective chart review of children <18 years old who required ECMO at a tertiary level hospital. We collected TEG^®^–PM and conventional coagulation tests data. We also collected demographic, medications, blood products administered, and clinical outcome data. We defined severe platelet dysfunction as <50% aggregation in response to an agonist.

**Results:**

We identified 24 out of 46 children on ECMO, who had TEG^®^–PM performed during the study period. We found the incidence of severe bleeding was 42% and mortality was 54% in our study cohort. In all samples measured, severe qualitative platelet dysfunction was more common for adenosine diphosphate (ADP)-mediated aggregation (92%) compared to arachidonic acid (AA)-mediated aggregation (75%) (*p* = 0.001). Also, ADP-mediated percent of platelet aggregation was significant lower than AA-mediated platelet aggregation [15% (interquartile range, IQR 2.8–48) vs. 49% (IQR 22–82.5), *p* < 0.001]. There was no difference in kaolin-activated heparinase TEG^®^ parameters between the bleeding group and the non-bleeding group. Only absolute platelet count and TEG^®^–PM had increased predictive value on receiver operating characteristics analyses for severe bleeding and mortality compared to activated clotting time.

**Conclusion:**

We found frequent and severe qualitative platelet dysfunction on TEG^®^–PM testing in children on ECMO. Larger studies are needed to determine if the assessment of qualitative platelet function by TEG^®^–PM can improve prediction of bleeding complications for children on ECMO.

## Introduction

Extracorporeal membrane oxygenation (ECMO) provides life-saving support to critically ill children with severe respiratory or cardiac failure when conventional therapies are inadequate. Approximately 2,000 children receive ECMO support every year, and the frequency is steadily increasing (1). Children on ECMO have high mortality rates between 35 and 50%, and most deaths are related to thromboembolic or bleeding complications (1, 2). In a recent study, bleeding and thromboembolic complications have shown to decrease survival by 24% (2).

Extracorporeal membrane oxygenation-induced coagulopathy (EIC) is a dynamic and complex pathophysiologic state of hemostasis, which is not completely understood. EIC results from complex interactions between hemostatic, endothelial, and immune systems that are perturbed by the ECMO circuit and the patient’s underlying illness (3). Anticoagulation is essential to keep the ECMO circuit from clotting, but it increases the risk of bleeding especially if the underlying EIC is not corrected. Intensive hemostasis monitoring is required to assess the underlying EIC and the adequacy of anticoagulation to minimize bleeding complications. Activated clotting time (ACT) remains the primary test to monitor hemostasis and anticoagulation for children on ECMO (4). Many centers have been using additional tests including antithrombin (ATIII) activity and anti-factor Xa assays to assess the adequacy of anticoagulation (5). Many studies have reported a weak predictive value of these tests in a setting of worsening EIC (6–12).

Qualitative platelet dysfunction has shown to occur in children on ECMO and may play a role in EIC (13–15). Routine monitoring of absolute platelet count to guide platelet transfusion is frequently done on ECMO, but qualitative platelet function is seldom measured. Few centers have already incorporated functional tests, such as viscoelastic assays [thromboelastography (TEG^®^)/rotational thromboelastometry (ROTEM)] and platelet function assays [platelet aggregometry or TEG^®^–platelet mapping (TEG^®^–PM)] to improve hemostasis monitoring for children on ECMO (5). There is limited data regarding the frequency and magnitude of abnormalities detected by these measures in the pediatric population, what thresholds indicate the need for therapy, and what are the treatment goals (4, 16).

In this pilot study, our primary objective was to determine the frequency and magnitude of platelet dysfunction according to TEG^®^–PM. The secondary objective was to analyze the ability of conventional and functional hemostatic measures to predict severe bleeding and mortality. We hypothesized that qualitative platelet dysfunction according to TEG^®^–PM is common and severe in children on ECMO, and TEG^®^–PM measures are a better predictor of bleeding complications and mortality in comparison to conventional hemostatic measures.

## Materials and Methods

### Patient Population and Study Variables

We conducted a retrospective chart review of children <18 years of age who received ECMO support from 9/1/2011 to 12/31/2012 at St. Louis Children’s Hospital in the neonatal (NICU), cardiac (CICU), and pediatric intensive care units (PICU). All the patients on whom TEG^®^–PM was performed during ECMO support were included in the study cohort. For each of these patients, we collected (1) demographics (age, weight, gender, diagnosis, type and duration of ECMO support), (2) hemostasis data [ACT, prothrombin (PT), activated partial thromboplastin time (aPTT), platelet count, fibrinogen, antithrombin activity (ATIII), TEG^®^–PM parameters, blood products transfused within 24 h prior to the TEG^®^–PM sample, and dose of heparin infusion at the time of the TEG^®^–PM sample], (3) medications which may affect platelet function including inhaled nitric oxide (iNO), milrinone, and H_2_-blockers given in 24 h prior to the TEG^®^–PM sample, and (4) outcomes data (severe bleeding while on ECMO support and death prior to ICU discharge).

### Definitions

We defined severe bleeding as intracranial, pulmonary, or gastrointestinal bleeding that required >50% decrease of heparin infusion for ≥12 h or >20 cc/kg of packed red blood transfusion in 24 h. Severe platelet dysfunction was arbitrarily defined as <50% platelet aggregation in response to an agonist on TEG^®^–PM, since no standard or well-accepted definition exists.

### Thromboelastography with Platelet Mapping Testing

Thromboelastography–platelet mapping (TEG^®^ 5000, Haemonetics Corporation analyzer system, Braintree, MA, USA) is a whole blood viscoelastic assay that has been well described in the literature (13, 17). In brief, the maximum clot strength (MA_Thrombin_) is measured by a kaolin-activated whole blood sample treated with citrate. To measure the contribution of fibrin only (MA_Fibrin_) in the clot strength, a whole blood heparinized sample is used to remove thrombin effect, and Reptilase and Factor XIII (Activator F) are added to the sample to generate a fibrin cross-linked clot. To measure the contribution to clot strength by adenosine diphosphate (ADP) (MA_ADP_) or arachidonic acid (AA) (MA_AA_) platelet surface receptors, Activator F plus ADP or AA is added to a whole blood heparinized sample. To calculate the percent platelet aggregation, the following formula is used: [(MA_ADP or AA_ − MA_Fibrin_)/(MA_Thrombin_ − MA_Fibrin_) × 100]%.

We had to limit the study period from 9/1/2011 to 12/31/2012 as new institutional guidelines were put in place beyond this period for ECMO hemostasis management. During the study period, anticoagulation management and blood products administration were based on ACT and conventional hemostasis measures including hemoglobin, fibrinogen, INR, and platelet count in accordance to the Extracorporeal Life Support Organization (ELSO) guidelines (18). Anti-Xa, ATIII, TEG^®^, and TEG^®^–PM were performed in individual patients based on risk of bleeding, ongoing bleeding, need of blood products, and adequacy of anticoagulation assessed by the treating physician. We did not have a standardized anticoagulation or transfusion algorithm based on anti-Xa, TEG^®^, and TEG^®^–PM measures during the study period. Few patients had more than five TEG^®^ samples performed during the ECMO support. No more than three TEG^®^–PM samples were analyzed per patient to minimize the sampling bias. In patients who had more than three TEG^®^–PM performed, we only included the first sample, the last sample, and the sample collected in 24 h prior to a severe bleeding event or at the midpoint of the time on ECMO support for the analysis. One investigator was blinded to all results when TEG^®^–PM samples were chosen for analysis for patients with more than three samples drawn. To determine the association of ADP- or AA-mediated platelet aggregation and severe bleeding, only blood samples taken for TEG^®^–PM and conventional hemostatic tests prior to the severe bleeding event were analyzed. For mortality prediction, we analyzed all blood samples in non-survival and compared with all samples taken prior to coming off the ECMO support in survivors.

### Statistics

The primary objective of this pilot study was to determine the frequency and magnitude of platelet dysfunction. Our secondary objective was to compare the ability of TEG^®^–PM and conventional coagulation measures to predict both severe bleeding complications and mortality. To determine normality, we used histograms to compare the sample data to a normal probability curve and also applied Shapiro–Wilk test of normality. Data are presented as median and interquartile range (IQR). We applied Mann–Whitney *U* tests to non-normally distributed data and used *Z* value to determine effect size. We used Spearman’s rank correlation coefficient to test the correlation between platelet count and ADP- or AA-mediated platelet aggregation. We used forced entry forward stepwise logistic regression model to estimate predictive models of bleeding and mortality. We performed receiver operating characteristic (ROC) curve analyses to determine area under the curve (AUC) and its 95% confidence interval of both conventional and functional coagulation parameters to predict severe bleeding complications and mortality. The analyses were performed by using IBM^®^ and SPSS^®^ version 21. The Institutional Review Board of Washington University in St. Louis approved this study.

## Results

We identified 24 out of 46 consecutive children supported on ECMO for various indications, who had TEG^®^–PM performed during the study period from 9/1/2011 to 12/31/2012 (Table 1). A total of 57 TEG^®^–PM samples were analyzed from these 24 patients with a median (IQR) of 2 (1–3) samples per patient. Patients had a median age of 9 (1–70) months and had received ECMO support for a median of 8 (6–10) days. Eighteen (75%) patients received venoarterial (VA)-ECMO and 6 (25%) patients received venovenous (VV)-ECMO support. Severe bleeding occurred in 10 (42%) patients and 13 (54%) patients died before ICU discharge. All patients were on unfractionated heparin infusions with a median dose of 40 (IQR 30–50) units/kg/h at the time TEG^®^–PM samples were drawn. 79% (19) of patients were receiving ranitidine, 50% (12) iNO, and 46% (11) milrinone 24 h before at least one of the TEG^®^–PM samples. None of these patients received direct antiplatelet or antifibrinolytic agents.

**Table 1:** **Indications of ECMO support in the study group**.

Indication of ECMO		Number (*N* = 24)
**ARDS^a^**		**4**
Sepsis	2	
RSV^b^	1	
Cystic fibrosis	1	
**Pulmonary hypertension**		**4**
**Congenital diaphragmatic hernia**		**2**
**Myocarditis/dilated cardiomyopathy**		**3**
**Postoperative**		**11**
Heart transplant	3	
Single ventricular	3	
AVC^c^ defect	1	
Pulmonary stenosis	1	
Aortic valve replacement	1	
Heart lung transplant	1	
Lung transplant	1	

*^a^Acute respiratory distress syndrome*.

*^b^Respiratory syncytial virus*.

*^c^Atrioventricular canal defect*.

**Figure 1:**
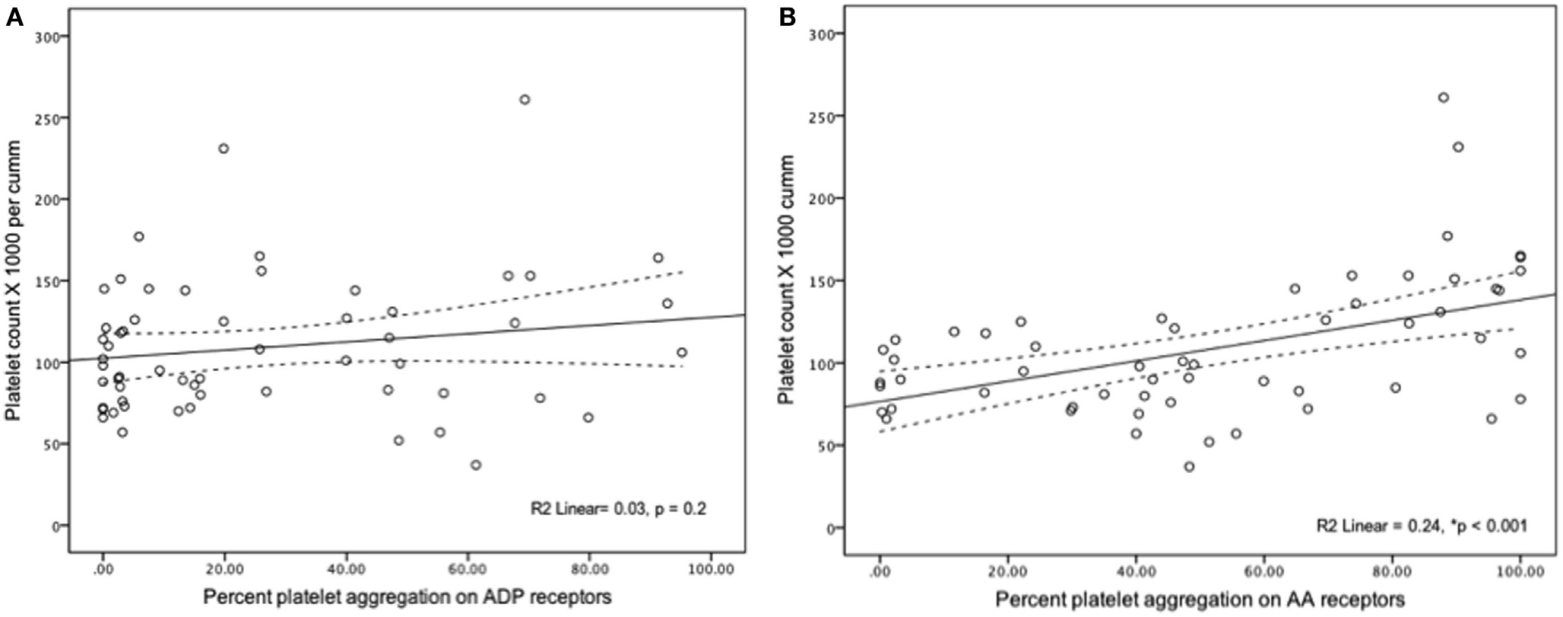
**(A,B)** Correlation of platelet aggregation and platelet count.

In all samples measured, severe platelet dysfunction (<50% aggregation in a response to an agonist) was more common for ADP-mediated aggregation (92%) compared to AA-mediated aggregation (75%) (*p* = 0.001). ADP-mediated percent of platelet aggregation was lower when compared to AA-mediated platelet aggregation [15% (IQR 2.8–48) vs. 49% (IQR 22–82.5), *p* < 0.001]. Platelet count had statistically significant but weak correlation with AA-mediated PA (*r*^2^ = 0.24, *p* = 0.001) and no correlation with ADP-mediated aggregation (*r*^2^ = 0.03, *p* = 0.2) (Figure 1). When analyzed individually, the use of the following medications within 24 h of sampling were not significantly associated with ADP-mediated aggregation: iNO vs. no iNO [12% (3–26) vs. 22% (2–50) (*p* = 0.44)], milrinone vs. no milrinone [25.7% (4–53) vs. 9.3% (1.7–44) (*p* = 0.14)], and ranitidine vs. no ranitidine [19.8% (5–48) vs. 3.2% (1–47) (*p* = 0.07)].

Univariate analysis revealed that reduced AA-mediated aggregation was associated with severe bleeding and reduced ADP-mediated aggregation was associated with mortality (Tables 2 and 3). On the multivariable logistics forced entry of ACT, ADP-mediated aggregation, AA-mediated platelet aggregation, and platelet count in a forward stepwise model for severe bleeding and mortality, only low platelet count was associated with increased odds of mortality (odds ratio 0.983, CI 0.968–0.999, *p* = 0.04).

**Table 2:** **Comparison of clinical and coagulation variables between no bleeding and severe bleeding group**.

Parameter	No severe bleeding^c^ (*n* = 14)	Severe bleeding^c^ (*n* = 10)	*p*-Value	Effect size *r* value
**Clinical variables**				
Age (months)	9 (0.8–12)	48 (2.7–90)	0.40	−0.17
Female gender [*n* (%)]	9 (64)	5 (50)	0.57	0.10
ECMO VA [*n* (%)]	10 (71)	9 (90)	0.17	0.24
Number of TEG samples per patient	2 (1–3)	2 (1–3)	0.9	–
Duration of ECMO support (days)	7 (6–10)	8 (5–9)	0.99	−0.002
Heparin dose (U/kg/h)	40 (30–50)	30 (20–48)	0.04*	−0.42
Survival [*n* (%)]	10 (72%)	3 (30%)	0.003*	0.41
**Medications**^a^				
iNO	6 (43%)	6 (60%)	0.16	–
Milrinone	6 (43%)	5 (50%)	0.78	–
Ranitidine	10 (71%)	9 (90%)	0.27	–
**Conventional coagulation tests**				
INR	1.29 (1.11–1.63)	1.33 (1.19–1.62)	0.20	−0.26
aPTT (s)	150 (119–150)	140 (87–150)	0.43	−0.18
ACT (s)	186 (166–196)	185 (164–198)	0.43	−0.16
Platelet count × 1000 per cumm	118 (80–144)	88 (76–120)	0.11	−0.32
Fibrinogen (mg/dl)	222 (192–358)	256 (201–346)	0.58	−0.03
Antithrombin activity (%)	44 (25–73)	40 (14–86)	0.80	
**TEG**^®^**–PM parameters**				
Reaction time	6.3 (5.4–8.1)	6.9 (6.3–8.5)	0.34	−0.20
*K* time	2 (1.7–2.7)	2.2 (1.5–2.7)	0.90	−0.02
Maximum amplitude (MA)	57 (51–62)	57 (54.3–62)	0.39	−0.18
Percent lysis at 30 min	0 (0–1)	0 (0.1)	0.70	−0.07
ADP-mediated platelet aggregation	26.7 (2.9–67.7)	9.3 (2.25–23.3)	0.09	−0.34
AA-mediated platelet aggregation	63.4 (41.5–95.7)	40 (14.2–67.5)	0.03*	−0.45
**Blood products transfused**^b^				
pRBC transfusion (mL/kg)	9 (0–20)	20 (0–30)	0.10	−0.34
PC transfusion (mL/kg)	0 (0–15)	8 (0–17.5)	0.86	−0.03
FFP transfusion (mL/kg)	0 (0–15)	4 (0–15)	0.72	−0.07

*^a^Medication prescribed within 24 h prior to the TEG–PM sample drawn*.

*^b^Blood product transfused within 24 h prior to the TEG–PM sample drawn*.

*^c^Data are represented as median (interquartile range) or number (percentage) as applicable*.

*p < 0.05 considered significant.

**Table 3:** **Comparison of clinical and coagulation variables between survival and non-survival group**.

Parameter	Survival^b^ (*n* = 13)	Non-survival^b^ (*n* = 11)	*p*-Value	Effect size *r* value
**Clinical variables**				
Age (months)	9 (7–66)	4 (0.6–70)	0.49	−0.15
Female gender [*n* (%)]	7 (54)	6 (54)	0.79	0.06
ECMO VA [*n* (%)]	8 (62)	10 (90)	0.02*	0.32
Number of TEG samples per patient	2 (1–3)	2 (1–3)	0.9	–
Duration of ECMO support (days)	7 (5–10)	9 (7–12)	0.14	−0.33
Heparin dose (U/kg/h)	40 (30–45)	40 (30–40)	0.66	−0.10
**Conventional coagulation tests**				
INR	1.29 (1–1.48)	1.36 (1.17–1.63)	0.14	−0.32
aPTT (s)	150 (112–150)	119 (74–150)	0.16	−0.31
ACT (s)	186 (179–196)	183 (172–199)	0.86	−0.04
Platelet count × 1000 per cumm	124 (92–151)	85 (71–112)	0.002*	−0.67
Fibrinogen (mg/dl)	238 (210–331)	273 (192–396)	0.76	−0.07
Antithrombin activity (%)	72 (35–80)	36 (17–72)	0.09	–
**TEG**^®^**–PM parameters**				
Reaction time	6.6 (5.4–8)	6.7 (5.7–7.6)	0.92	−0.02
*K* time	2 (1.7–2.4)	2 (1.5–2.7)	0.87	−0.03
Maximum amplitude (MA)	57 (52–62)	58 (54–62)	0.80	−0.06
Percent lysis at 30 min	0 (0–1)	0 (0–1)	0.56	−0.13
ADP-mediated platelet aggregation	26 (7–65)	5 (1–43)	0.03*	−0.50
AA-mediated platelet aggregation	74 (22–96)	41 (20–66)	0.05	−0.44
**Blood products transfused**^a^				
pRBC transfusion (mL/kg)	10 (0–24)	10 (10–23)	0.92	−0.02
PC transfusion (mL/kg)	0 (0–11.5)	10 (0–15)	0.04*	−0.44
FFP transfusion (mL/kg)	0 (0–10)	10 (0–15)	0.04*	−0.45

*^a^The amount of blood product transfused within 24 h prior to the TEG–PM sample drawn*.

*^b^Data are represented as median (interquartile range) or number (percentage) as applicable*.

*p < 0.05 considered significant.

**Figure 2:**
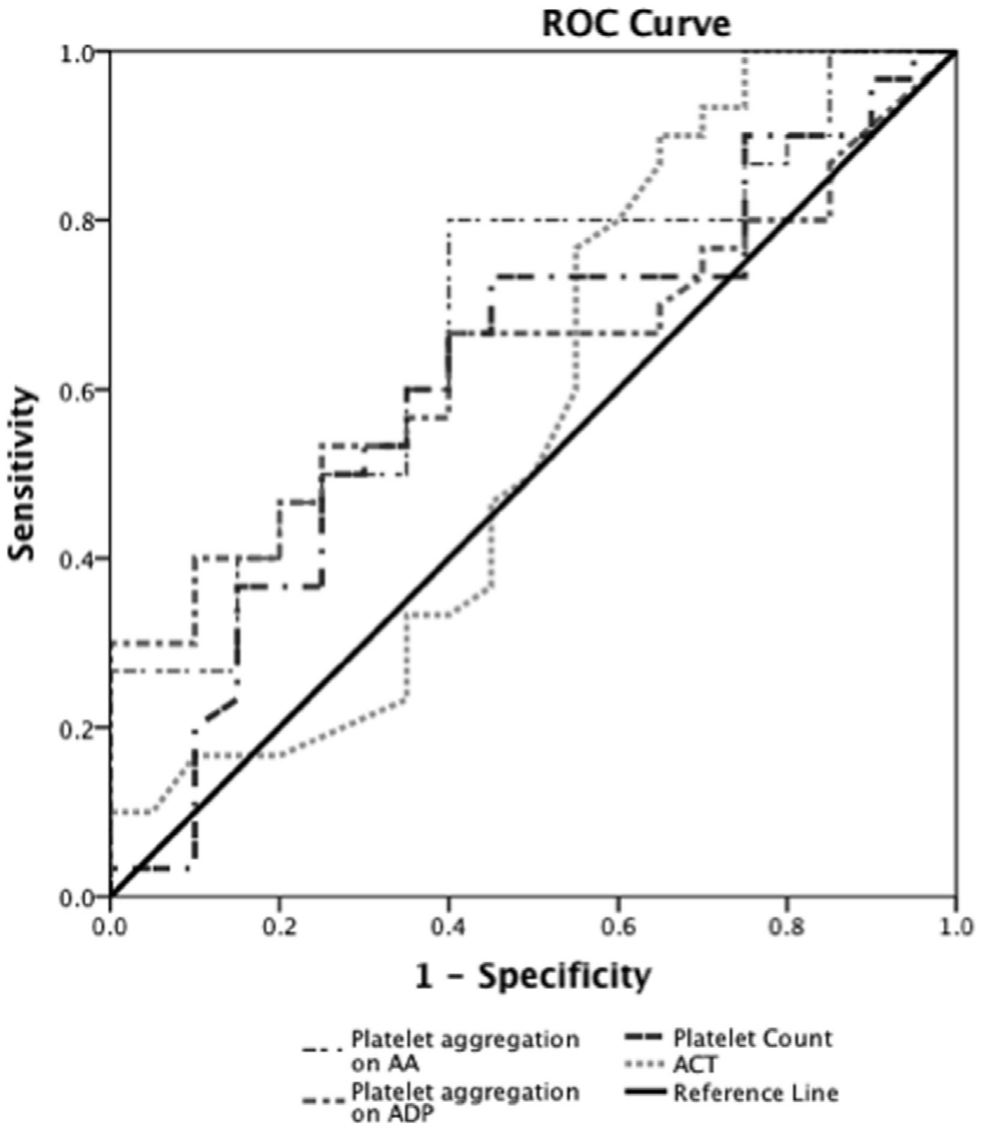
**Receiver operator curve analyses for hemostatic parameters to predict severe bleeding**. On ROC analyses (*n* = 24), we found that the AUC for ACT was lowest at 0.56 (0.39–0.75, *p* = 0.42) and platelet count was 0.61 (0.45–0.78, *p* = 0.16). The AUC for ADP-mediated platelet aggregation 0.64 (0.48–0.79, *p* = 0.11) and AA-mediated platelet aggregation 0.68 (0.53–0.83, *p = 0.03).*p < 0.05 considered significant.

**Figure 3:**
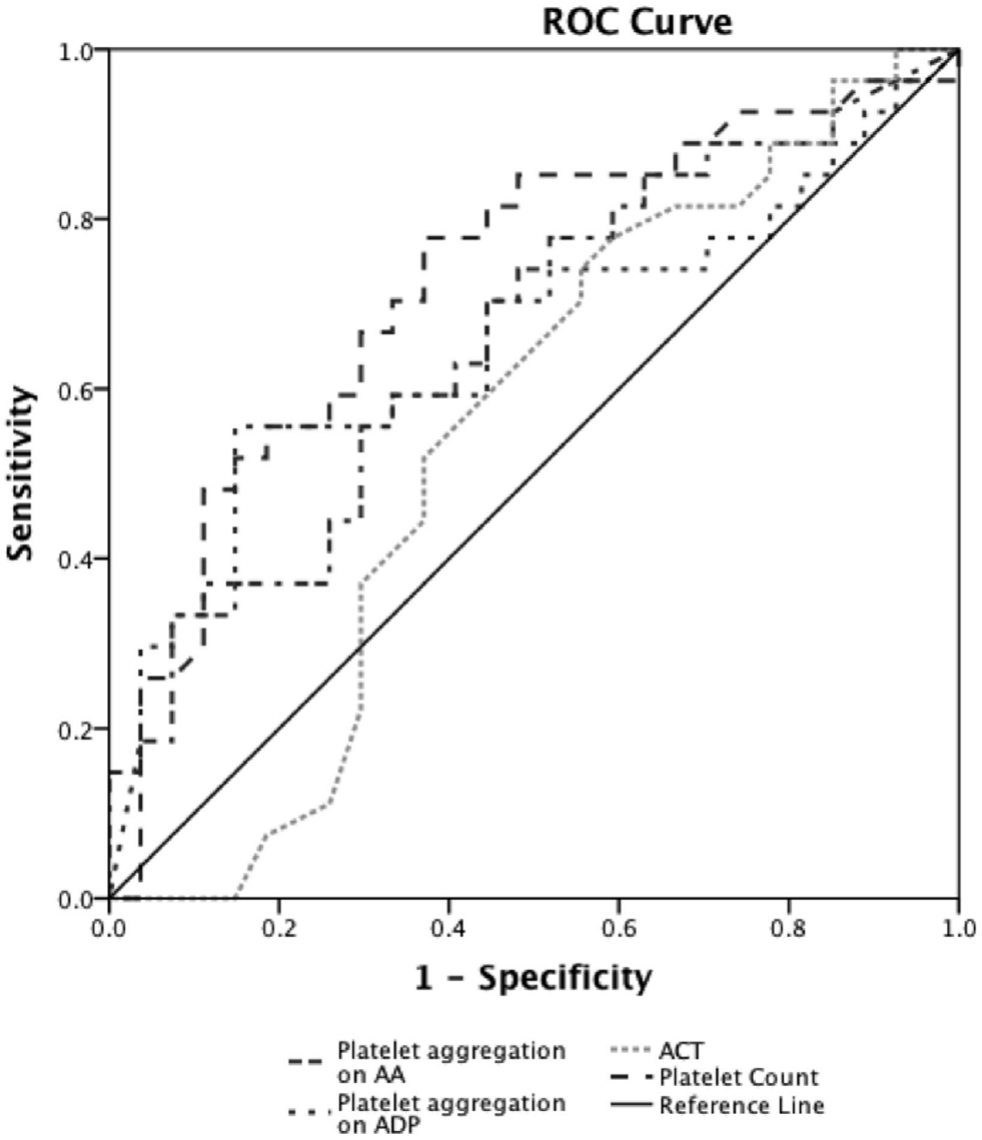
**Receiver operator curve analyses for hemostatic parameters to predict mortality**. On ROC analyses (*n* = 24), we found that the AUC for ACT (0.51, 0.35–0.67, *p* = 0.9) was the lowest compared to quantitative platelet count (0.73, 0.59–0.86, *p = 0.004) and TEG^®^–PM parameters of qualitative platelet function [ADP-mediated platelet aggregation (0.67, 0.52–0.8, *p = 0.03) and AA-mediated platelet aggregation (0.65, 0.50–0.80, *p* = 0.06)]. *p < 0.05 considered significant.

Prediction of severe bleeding based on ROC curves indicated that the AUC and 95% Confidence interval (95% CI) for the ACT was lowest at 0.56 (0.39–0.75, *p* = 0.42) and platelet count was 0.61 (0.45–0.78, *p* = 0.16). The AUC for ADP-mediated platelet aggregation 0.64 (0.48–0.79, *p* = 0.11) and AA-mediated platelet aggregation 0.68 (0.53–0.83, *p = 0.03) (Figure 2). Prediction of mortality based on ROC revealed that the AUC for ACT (0.51, 0.35–0.67, *p* = 0.9) was the lowest compared to quantitative platelet count (0.73, 0.59–0.86, *p = 0.004) and qualitative markers of platelet function [ADP-mediated platelet aggregation (0.67, 0.52–0.8, *p = 0.03) and AA-mediated platelet aggregation (0.65, 0.50–0.80, *p* = 0.06)] (Figure 3).

## Discussion

To the best of our knowledge, this study is the first to report the frequency and magnitude of qualitative platelet function according to TEG^®^–PM for children on ECMO. We found frequent (more than 75% of patients) and severe qualitative platelet dysfunction on TEG^®^–PM in our patient cohort. The platelet dysfunction found on TEG^®^–PM measures in our patient cohort was more pronounced compared to the routine TEG^®^–PM evaluation of healthy adults and children with congenital heart defect before cardiopulmonary bypass (CPB) (13, 19). The qualitative platelet dysfunction was more common and pronounced to ADP-mediated aggregation compared to AA-mediated aggregation. ADP is stored in dense granules and released upon platelet activation. The higher incidence of decrease in ADP-mediated platelet aggregation in our cohort may be related to weak agonist action of ADP and possible depletion of the stored ADP in the circulating platelets. Despite severe qualitative platelet dysfunction on TEG^®^–PM measures, we found that the maximum amplitude (MA), a surrogate of qualitative platelet function on kaolin-activated heparinase TEG^®^ measures, was at the lower end of normal range of healthy children in our cohort (20). One possible explanation for this observation is a compensatory supranormal fibrinogen response via glycoprotein IIb/IIIa-mediated platelet aggregation and thrombin response via protease-activated receptor (PAR)-mediated platelet aggregation to overcome decrease in ADP- and AA-mediated platelet aggregation (21, 22). Also, we found that *R* time, *K* time, and Lysis at 30 min on kaolin-activated heparinase TEG^®^ were within normal range for age in our cohort (20). In this small pilot study, platelet count and TEG^®^–PM measures were a better predictor of severe bleeding and mortality.

Qualitative platelet dysfunction has been described by using various assays in children requiring CPB or ECMO support (13–15). Chueng et al. have reported a time-dependent decrease in platelet aggregation measured by using whole blood platelet-ionized calcium lumi-aggregometer, despite repeated platelet transfusions to maintain platelet counts >100 × 10^3^/mm^3^ during ECMO support (14). Robinson et al. have shown decreased platelet aggregation by whole blood platelet lumi-aggregometry in response to collagen, ADP, and ristocetin stimulation within 15 min of cannulation for ECMO (15). They have also observed persistent platelet dysfunction even after platelet transfusions in their cohort (15). Weitzel et al. have reported impaired platelet aggregation in response to collagen, ADP, and AA by TEG^®^–PM measures in children with congenital heart defect requiring CPB (13). They found that TEG^®^–PM measures had sensitivity of 83% and specificity of 68% in predicting postoperative bleeding (13). Nair et al. have reported 50–72% incidence of platelet dysfunction in adult patients on ECMO by using Multiplate^®^ analyzer for ADP and ristocetin (23). Many medications, including iNO, milrinone, H_2_ receptor blockers, prostaglandins, sildenafil, and antiplatelet agents, which are frequently used in patients on ECMO can alter qualitative platelet dysfunction (24–27). Limited experimental and clinical data studying the effect of these medications on qualitative platelet function in children on ECMO are present (28–30). There was no significant association between the use of iNO, milrinone, and ranitidine, and ADP-mediated platelet aggregation in our cohort. This observation might be due to the high incidence and magnitude of platelet dysfunction on ADP-mediated aggregation and limited power to detect any difference in our cohort. It is difficult to make any comparison between the studies we discussed as most of these, including ours, are limited by sample size, heterogeneous study population, the timing of sampling, and different tests being used to measure the qualitative platelet function. These studies suggest frequent qualitative platelet dysfunction in children on ECMO, which is independent of absolute platelet count. The evaluation of qualitative platelet dysfunction by newer point-of-care platelet function assays may have a potential to enhance the hemostasis monitoring in children on ECMO. This can be challenging due to lack of an established gold standard to compare newer point-of-care platelet function assays and limited clinical experience and validation of these assays in children on ECMO (31, 32).

ECMO-induced coagulopathy is a dynamic pathophysiologic state of hemostasis that results from complex alterations and interactions of coagulation, fibrinolysis, platelets, endothelium and immune systems (3, 33, 34). Many factors contribute to EIC including the ECMO circuit, the patient’s underlying illness, anticoagulation agents, medications, and blood products administration (3, 34, 35). These factors restrict the ability of any particular single coagulation test to accurately identify bleeding risk; hence, our ability to treat patients at risk of EIC and resultant persistently high bleeding and thromboembolic complications rate in children on ECMO (2). Many centers have started incorporating multiple tests including viscoelastic (TEG^®^/ROTEM^®^) and platelet function assays (TEG^®^–PM and Impedance aggregometry) in the hemostasis evaluation to be able to improve the identification of patients at risk of EIC (3, 5). Use of these assays in adults undergoing CPB or with major trauma has shown to improve outcome, minimize blood product transfusion, and reduce the cost of treatment (36, 37). There are limited studies on efficacy and threshold parameters for these assays in for children on ECMO (38–40). Interestingly, we found that kaolin-activated heparinase TEG^®^ measures (reflective of the underlying hemostasis potential) were within normal range for age and also comparable between the bleeding and the non-bleeding patients or the surviving and the non-surviving. These observations may be due to the correction of TEG^®^ measures by frequent blood products administration and/or poor sensitivity of TEG^®^ measures to detect the EIC related to endothelial and qualitative platelet dysfunction. Comprehensive hemostasis evaluation is desirable for children on ECMO, but there are many challenges to such approach. Some of these challenges are limited data on age-related variations on the results of functional assays, need for frequent and large volume blood sampling, requirement of specialized technical support, interpretation requires additional training and expertise, can be expensive and resource intensive, inadequate data to support treatment threshold, and finally, no data to support clinical efficacy.

Our study was limited by its small sample size, heterogeneity of patients, and lack of specific time points that could affect patients’ hemostatic state. The above limitations preclude assessment of the temporal relationship between platelet dysfunction and duration of ECMO support and limit the power to assess the association between platelet dysfunction and clinical outcomes. The high incidence of platelet dysfunction in our cohort could be more than the typical ECMO patients due to selection of patients with high risk of bleeding in our cohort. We were not able to determine the effect of blood products administration on platelet dysfunction due to lack of TEG^®^–PM-guided transfusion protocol during the study period. Despite above limitations, this study provides useful information regarding frequency and magnitude of platelet dysfunction, which will help estimate sample size for a well-designed multicenter study to determine the clinical relevance of TEG^®^–PM analysis in this cohort of patients.

## Conclusion

We found frequent and severe qualitative platelet dysfunction on TEG^®^–PM testing in children on ECMO. Larger studies are required to determine if the assessment of qualitative platelet function by TEG^®^–PM can improve prediction of bleeding complications for children on ECMO.

## Author Contributions

AS: study design, data collection, data analyses, and manuscript writing. A Said: study design, IRB, and manuscript review. MH: data analyses, manuscript writing and review. AG: data analyses, manuscript writing and review. UB: study design, manuscript review. PE: study design, data analyses, and manuscript review. BG: critical manuscript review and editing. PS: overall study design, data analyses, and manuscript writing, editing, and review.

## Conflict of Interest Statement

Philip C. Spinella declares having received research support from Haemonetics: only reagents and supplies. No honorariums or consulting fees. The remaining authors declare that the research was conducted in the absence of any commercial or financial relationships that could be construed as a potential conflict of interest.

## References

[1] Paden ML Conrad SA Rycus PT Thiagarajan RR Registry E. Extracorporeal Life Support Organization registry report 2012. ASAIO J (2013) 59(3):202–10. 10.1097/MAT.0b013e3182904a52 23644605

[2] Dalton HJ Garcia-Filion P Holubkov R Moler FW Shanley T Heidemann S Association of bleeding and thrombosis with outcome in extracorporeal life support*. Pediatr Crit Care Med (2015) 16(2):167–74. 10.1097/PCC.0000000000000317 25647124 PMC4605822

[3] Saini A Spinella PC. Management of anticoagulation and hemostasis for pediatric extracorporeal membrane oxygenation. Clin Lab Med (2014) 34(3):655–73. 10.1016/j.cll.2014.06.014 25168949

[4] Bembea MM Schwartz JM Shah N Colantuoni E Lehmann CU Kickler T Anticoagulation monitoring during pediatric extracorporeal membrane oxygenation. ASAIO J (2013) 59(1):63–8. 10.1097/MAT.0b013e318279854a 23263338 PMC3532578

[5] Bembea MM Annich G Rycus P Oldenburg G Berkowitz I Pronovost P. Variability in anticoagulation management of patients on extracorporeal membrane oxygenation: an international survey. Pediatr Crit Care Med (2013) 14(2):e77–84. 10.1097/PCC.0b013e31827127e4 23287906 PMC3567253

[6] Owings JTPM Gosselin RC Ireland K Jahr JS Larkin EC. Anticoagulation of children undergoing cardiopulmonary bypass is overestimated by current monitoring techniques. Arch Surg (2000) 135(9):1042–7. 10.1001/archsurg.135.9.1042 10982508

[7] Despotis GJ Avidan MS Hogue CW Jr. Mechanisms and attenuation of hemostatic activation during extracorporeal circulation. Ann Thorac Surg (2001) 72(5):S1821–31. 10.1016/S0003-4975(01)03211-8 11722116

[8] Rojas A Vojnovic B Johns H Joiner MC Martindale C Fowler JF Radiosensitisation in normal tissues with oxygen, carbogen or nicotinamide: therapeutic gain comparisons for fractionated X-ray schedules. Radiother Oncol (1996) 39(1):53–64. 10.1016/0167-8140(95)01678-3 8735494

[9] Guzzetta NA Bajaj T Fazlollah T Szlam F Wilson E Kaiser A A comparison of heparin management strategies in infants undergoing cardiopulmonary bypass. Anesth Analg (2008) 106(2):419–25. 10.1213/01.ane.0000297290.03501.db 18227295

[10] Khaja WA Bilen O Lukner RB Edwards R Teruya J. Evaluation of heparin assay for coagulation management in newborns undergoing ECMO. Am J Clin Pathol (2010) 134(6):950–4. 10.1309/AJCPGVD62LKKVDLH 21088159

[11] Green TP Isham-Schopf B Steinhorn RH Smith C Irmiter RJ. Whole blood activated clotting time in infants during extracorporeal membrane oxygenation. Crit Care Med (1990) 18(5):494–8. 10.1097/00003246-199005000-00006 2328593

[12] Esper SA Levy JH Waters JH Welsby IJ. Extracorporeal membrane oxygenation in the adult: a review of anticoagulation monitoring and transfusion. Anesth Analg (2014) 118(4):731–43. 10.1213/ANE.0000000000000115 24651227

[13] Weitzel NS Weitzel LB Epperson LE Karimpour-Ford A Tran ZV Seres T Platelet mapping as part of modified thromboelastography (TEG(R)) in patients undergoing cardiac surgery and cardiopulmonary bypass. Anaesthesia (2012) 67(10):1158–65. 10.1111/j.1365-2044.2012.07231.x 22809250

[14] Cheung PY Sawicki G Salas E Etches PC Schulz R Radomski MW. The mechanisms of platelet dysfunction during extracorporeal membrane oxygenation in critically ill neonates. Crit Care Med (2000) 28(7):2584–90. 10.1097/00003246-200007000-00067 10921599

[15] Robinson TM Kickler TS Walker LK Ness P Bell W. Effect of extracorporeal membrane oxygenation on platelets in newborns. Crit Care Med (1993) 21(7):1029–34. 10.1097/00003246-199307000-00018 8319460

[16] Nankervis CAPT Dysart KC. Assessing heparin dosing in neonates on venoarterial extracorporeal membrane oxygenation. ASAIO J (2007) 53:111–4. 10.1097/01.mat.0000247777.65764.b3 17237658

[17] Whiting D DiNardo JA. TEG and ROTEM: technology and clinical applications. Am J Hematol (2014) 89(2):228–32. 10.1002/ajh.23599 24123050

[18] Extracorporeal Life Support Organization. ELSO Guidelines for Cardiopulmonary Extracorporeal Life Support. Ann Arbor, MI: Extracorporeal Life Support Organization (2013). Available from: http://www.elsonet.org

[19] Bochsen L Wiinberg B Kjelgaard-Hansen M Steinbruchel DA Johansson PI. Evaluation of the TEG platelet mapping assay in blood donors. Thromb J (2007) 5:3. 10.1186/1477-9560-5-3 17311677 PMC1804261

[20] Chan KL Summerhayes RG Ignjatovic V Horton SB Monagle PT. Reference values for kaolin-activated thromboelastography in healthy children. Anesth Analg (2007) 105(6):1610–3. 10.1213/01.ane.0000287645.26763.be 18042858

[21] Ruggeri ZM Zarpellon A Roberts JR Mc Clintock RA Jing H Mendolicchio GL. Unravelling the mechanism and significance of thrombin binding to platelet glycoprotein Ib. Thromb Haemost (2010) 104(5):894–902. 10.1160/TH10-09-0578 20941453 PMC3810535

[22] Harrison P Wilbourn B Debili N Vainchenker W Breton-Gorius J Lawrie AS Uptake of plasma fibrinogen into the alpha granules of human megakaryocytes and platelets. J Clin Invest (1989) 84(4):1320–4. 10.1172/JCI114300 2677051 PMC329793

[23] Nair P Hoechter DJ Buscher H Venkatesh K Whittam S Joseph J Prospective observational study of hemostatic alterations during adult extracorporeal membrane oxygenation (ECMO) using point-of-care thromboelastometry and platelet aggregometry. J Cardiothorac Vasc Anesth (2015) 29(2):288–96. 10.1053/j.jvca.2014.06.006 25655210

[24] Scharf RE. Drugs that affect platelet function. Semin Thromb Hemost (2012) 38(8):865–83. 10.1055/s-0032-1328881 23111864

[25] Gudmundsdottir IJ McRobbie SJ Robinson SD Newby DE Megson IL. Sildenafil potentiates nitric oxide mediated inhibition of human platelet aggregation. Biochem Biophys Res Commun (2005) 337(1):382–5. 10.1016/j.bbrc.2005.09.060 16185664

[26] Tanriverdi S Koroglu OA Uygur O Balkan C Yalaz M Kultursay N. The effect of inhaled nitric oxide therapy on thromboelastogram in newborns with persistent pulmonary hypertension. Eur J Pediatr (2014) 173(10):1381–5. 10.1007/s00431-014-2325-3 24791933

[27] Kaushansky K Lichtman MA Beutler E Kipps TJ Seligsohn U Prchal JT Williams Hematology. 8th ed New York: The McGraw-Hill Companies, IBC (2010).

[28] Cheung PY Sawicki G Peliowski A Etches PC Schulz R Radomski MW. Inhaled nitric oxide inhibits the release of matrix metalloproteinase-2, but not platelet activation, during extracorporeal membrane oxygenation in adult rabbits. J Pediatr Surg (2003) 38(4):534–8. 10.1053/jpsu.2003.50116 12677560

[29] Rauch ED Stammers AH Mejak BL Vang SN Viessman TW. The effects of nitric oxide on coagulation during simulated extracorporeal membrane oxygenation. J Extra Corpor Technol (2000) 32(4):214–9. 11194058

[30] Tanaka H Tajimi K Miyajima Y Kazama M Kobayashi K. Effects of milrinone on platelet aggregation in swine with pulmonary hypertension. J Crit Care (2000) 15(3):113–8. 10.1053/jcrc.2000.16464 11011824

[31] Michelson AD Platelets. 3rd ed London:Academic Press (2013).

[32] Michelson AD. How platelets work: platelet function and dysfunction. J Thromb Thrombolysis (2003) 16(1–2):7–12. 10.1023/B:THRO.0000014586.77684.82 14760205

[33] Oliver WC. Anticoagulation and coagulation management for ECMO. Semin Cardiothorac Vasc Anesth (2009) 13(3):154–75. 10.1177/1089253209347384 19767408

[34] Peek GJ Firmin RK. The inflammatory and coagulative response to prolonged extracorporeal membrane oxygenation. ASAIO J (1999) 45(4):250–63. 10.1097/00002480-199907000-00003 10445729

[35] Spinella PC The blind physicians and the elephant on extracorporeal membrane oxygenation. Pediatr Crit Care Med (2013) 14(2):231–3. 10.1097/PCC.0b013e31827451ea 23388574

[36] Weber CF Gorlinger K Meininger D Herrmann E Bingold T Moritz A Point-of-care testing: a prospective, randomized clinical trial of efficacy in coagulopathic cardiac surgery patients. Anesthesiology (2012) 117(3):531–47. 10.1097/ALN.0b013e318264c644 22914710

[37] Da Luz LT Nascimento B Shankarakutty AK Rizoli S Adhikari NK Effect of thromboelastography (TEG(R)) and rotational thromboelastometry (ROTEM(R)) on diagnosis of coagulopathy, transfusion guidance and mortality in trauma: descriptive systematic review. Crit Care (2014) 18(5):518 10.1186/s13054-014-0518-9 25261079 PMC4206701

[38] Stammers AH Willett L Fristoe L Merrill J Stover T Hunt A Coagulation monitoring during extracorporeal membrane oxygenation: the role of thrombelastography. J Extra Corpor Technol (1995) 27(3):137–45. 10155358

[39] Stammers AH Bruda NL Gonano C Hartmann T Point-of-care coagulation monitoring: applications of the thromboelastography. Anaesthesia (1998) 53(Suppl 2):58–9. 10.1111/j.1365-2044.1998.tb15159.x 9659071

[40] Zavadil DP Stammers AH Willett LD Deptula JJ Christensen KA Sydzyik RT. Hematological abnormalities in neonatal patients treated with extracorporeal membrane oxygenation (ECMO). J Extra Corpor Technol (1998) 30(2):83–90. 10182118

